# Supplementary material for: Aspergillus niger as an efficient biological agent for separator sludge remediation: two-level factorial design for optimal fermentation
Source: PeerJ. 2024 Jul 15;12:e17151. doi: 10.7717/peerj.17151 (PMC11257062; doi:10.7717/peerj.17151)
Supplement: File S3 [file peerj-12-17151-s003.docx]

| 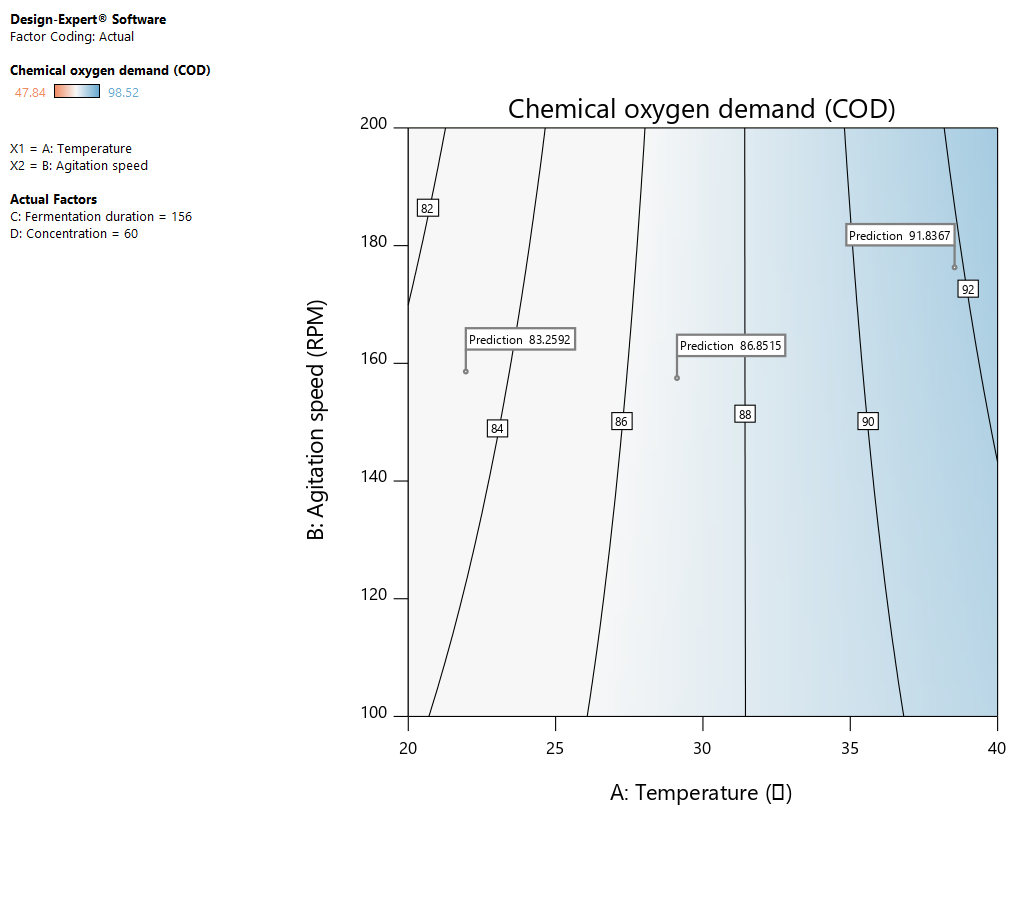 | 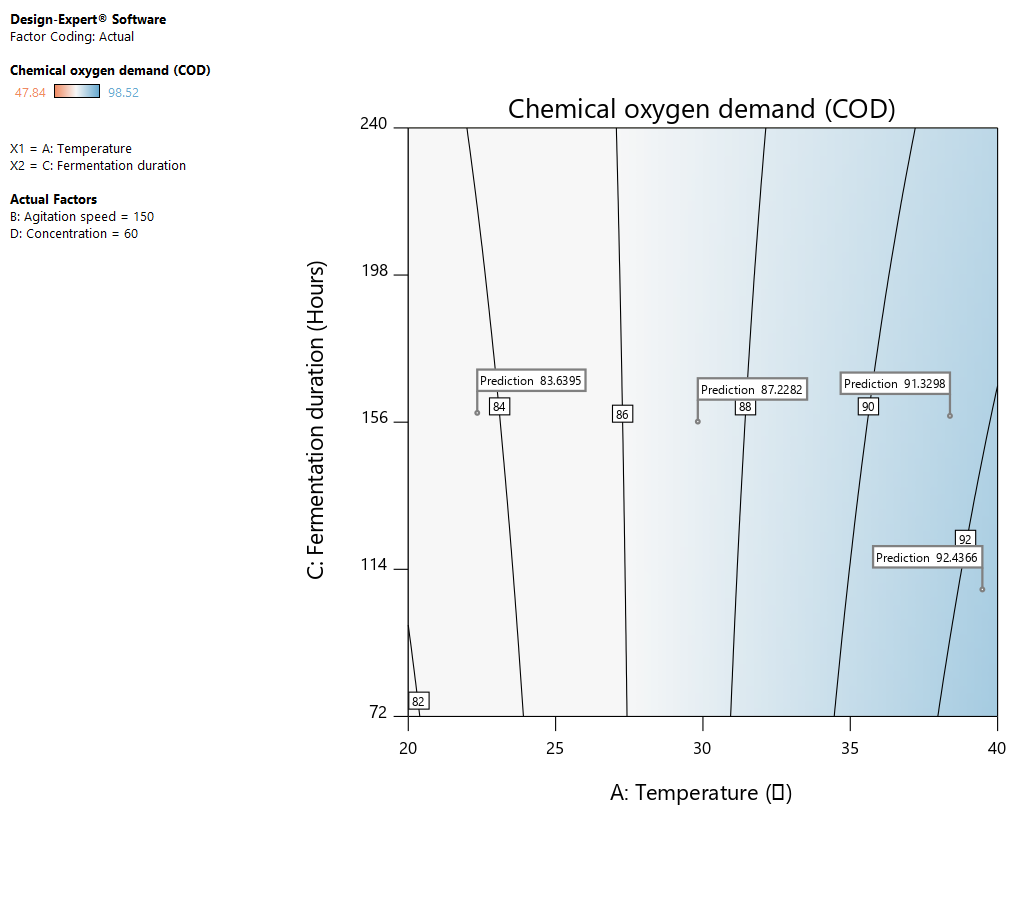 |
| --- | --- |
| 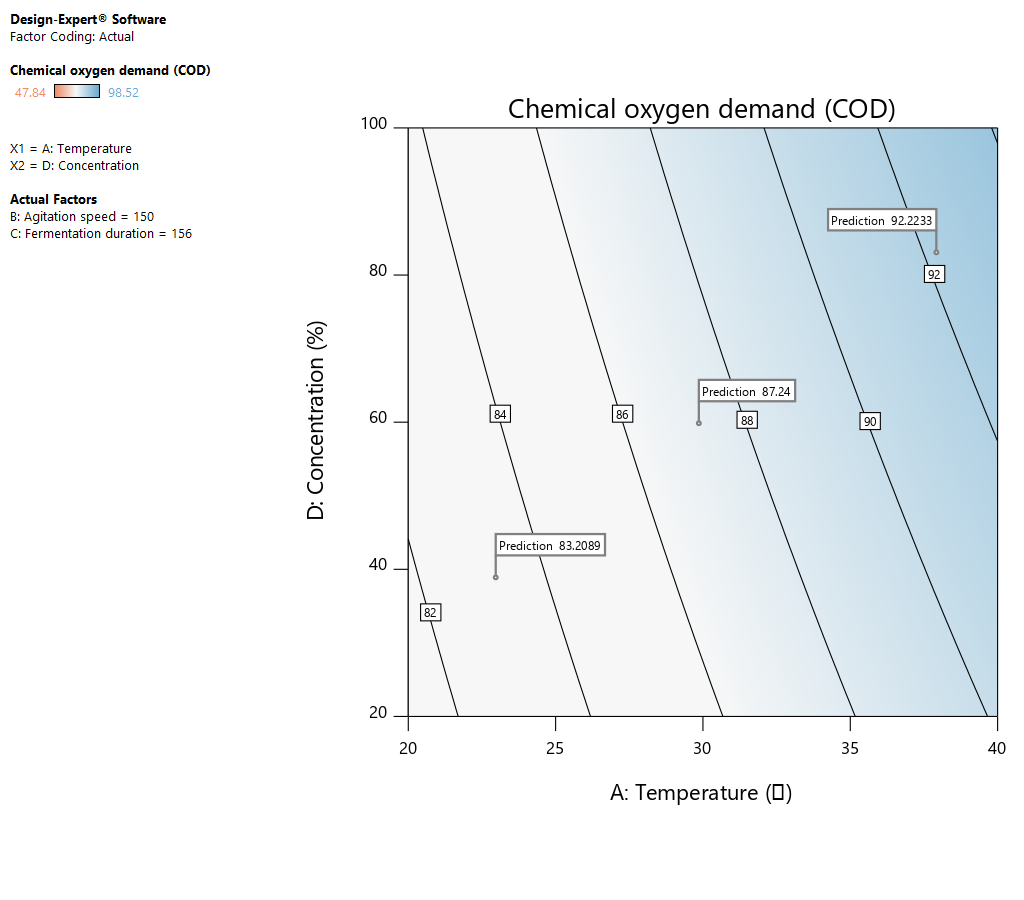 | 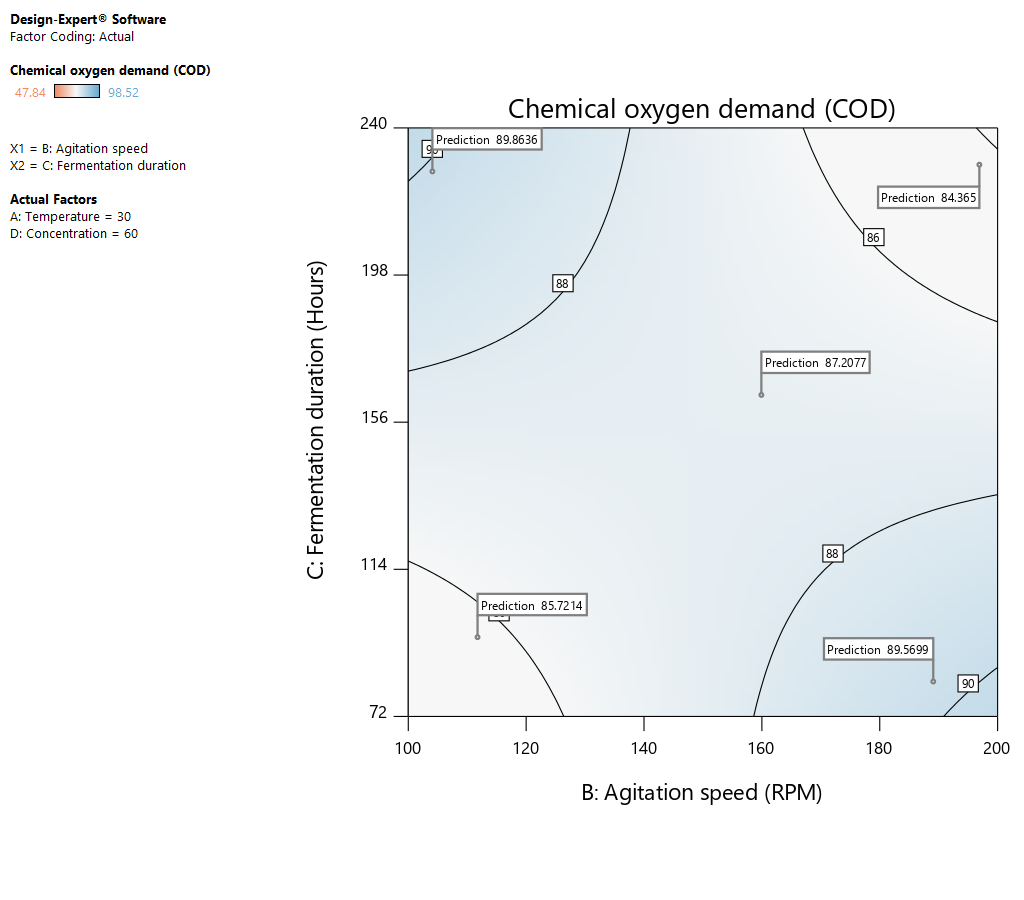 |
| 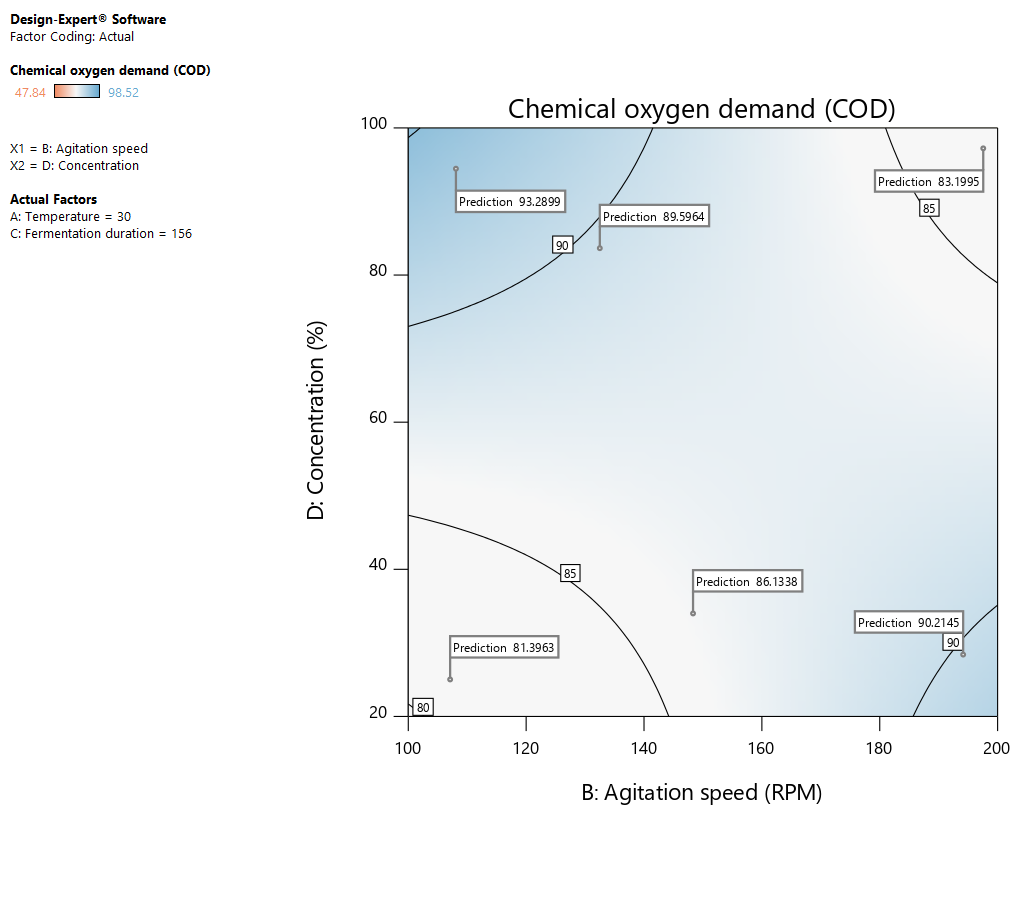 | 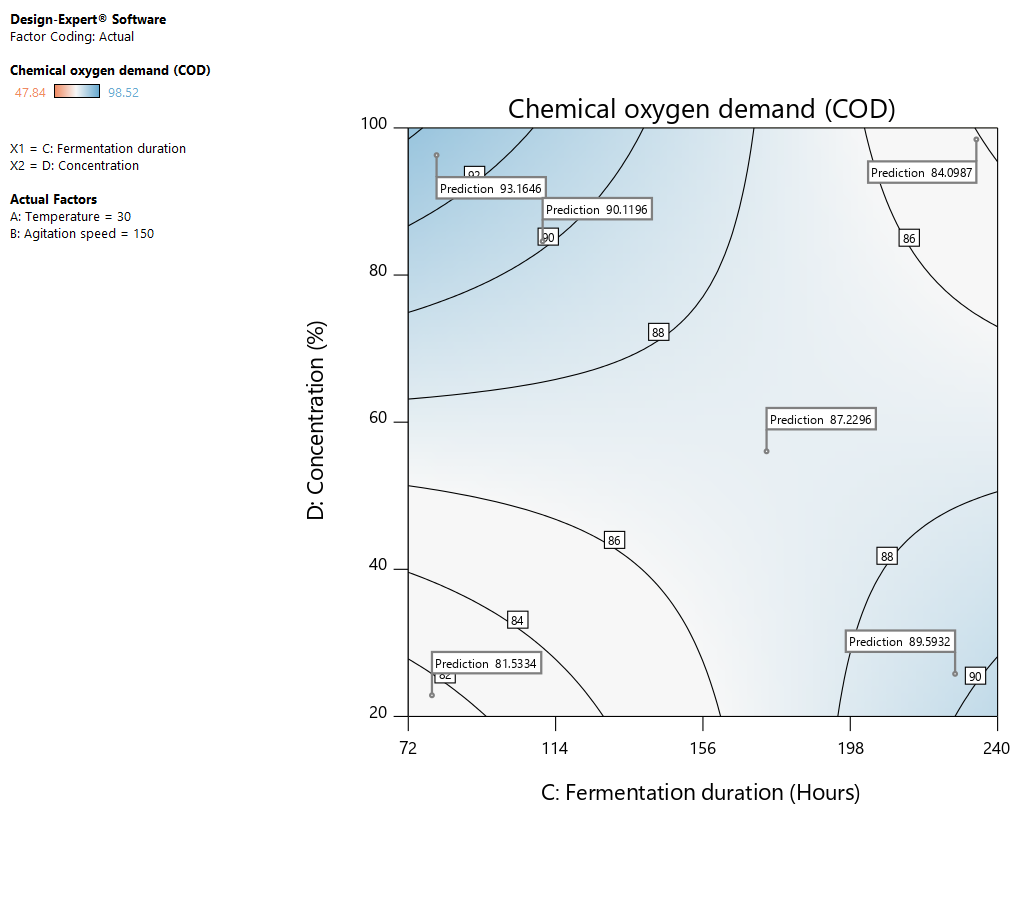 |

Contour plot of the effect of parameters on COD removal

| 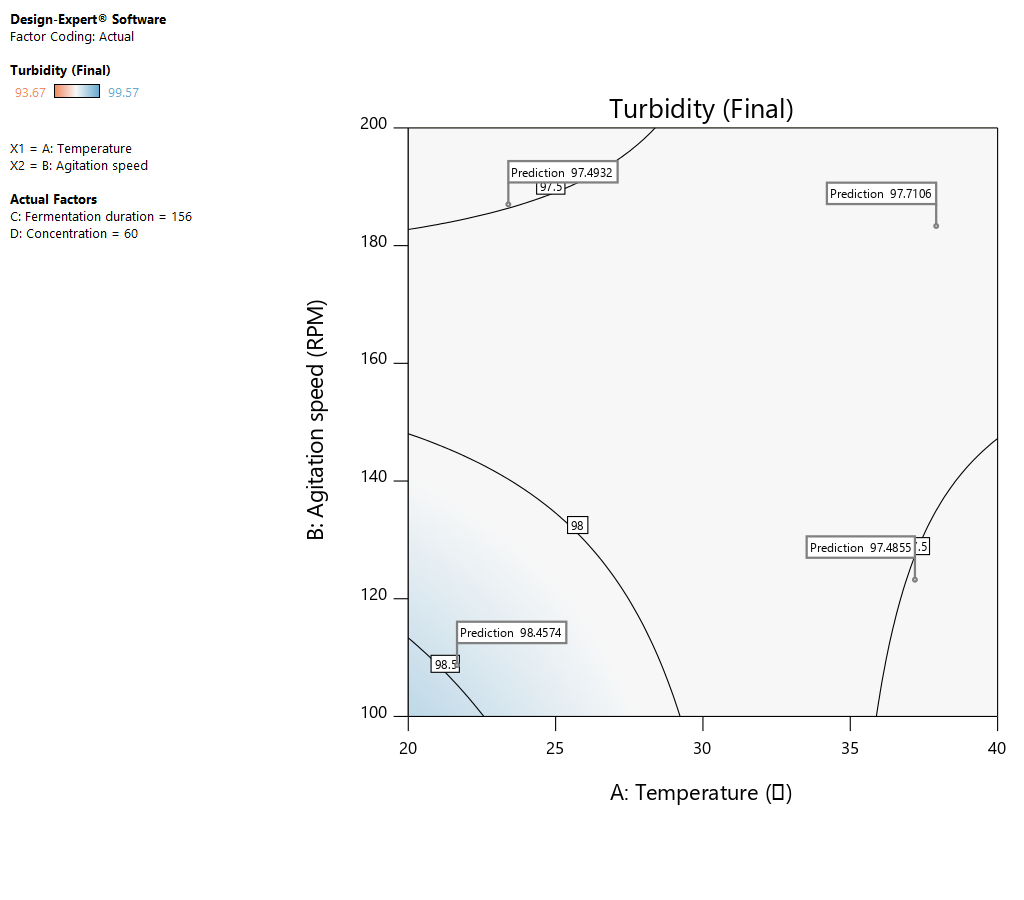 | 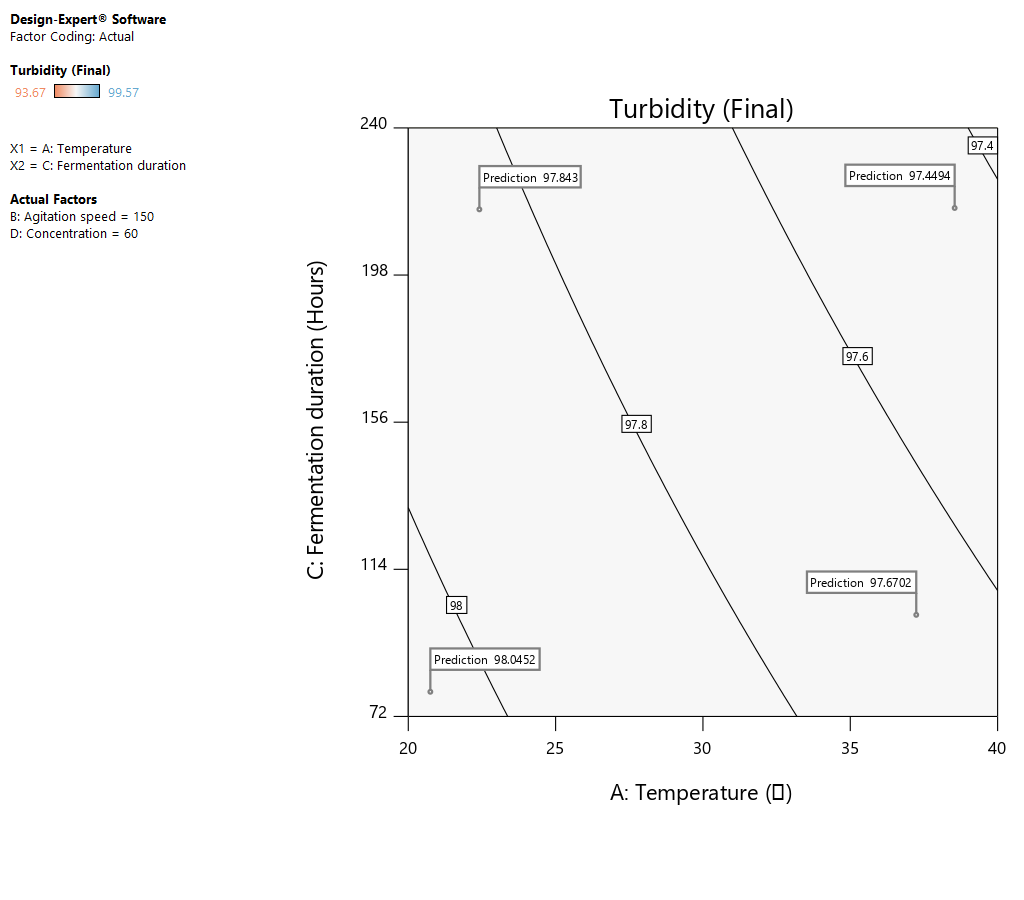 |
| --- | --- |
| 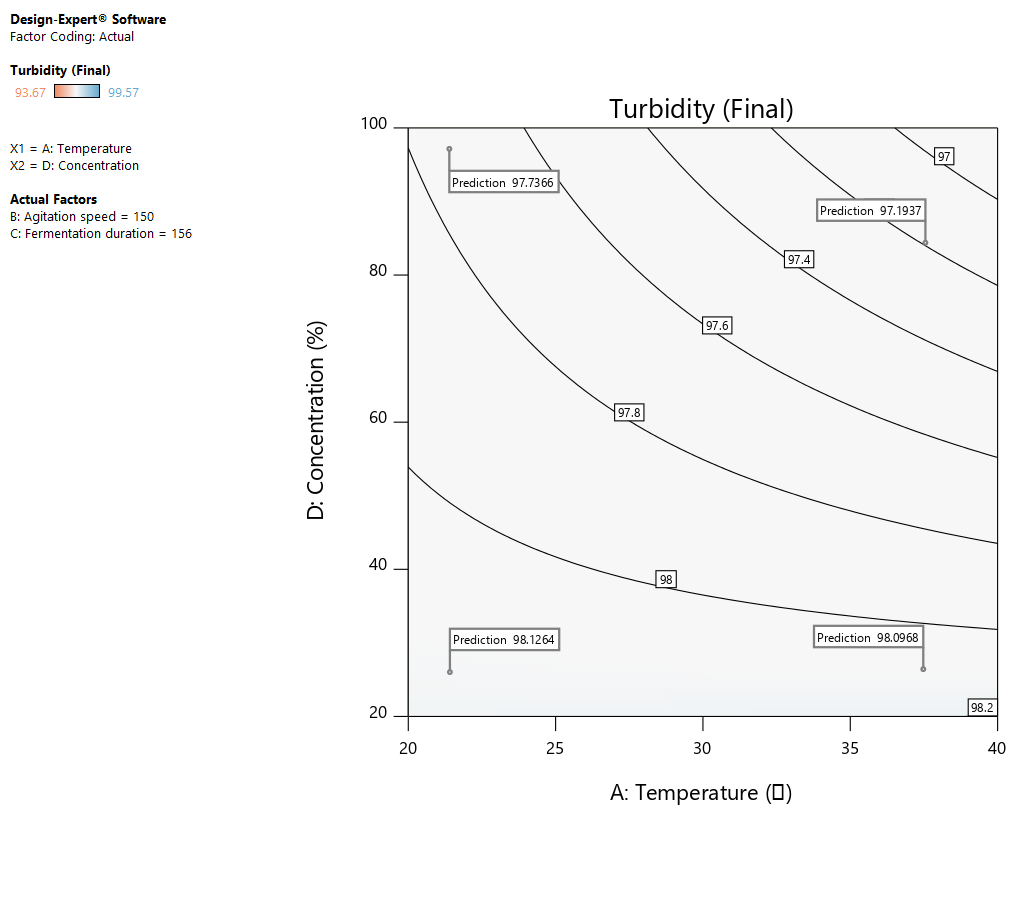 | 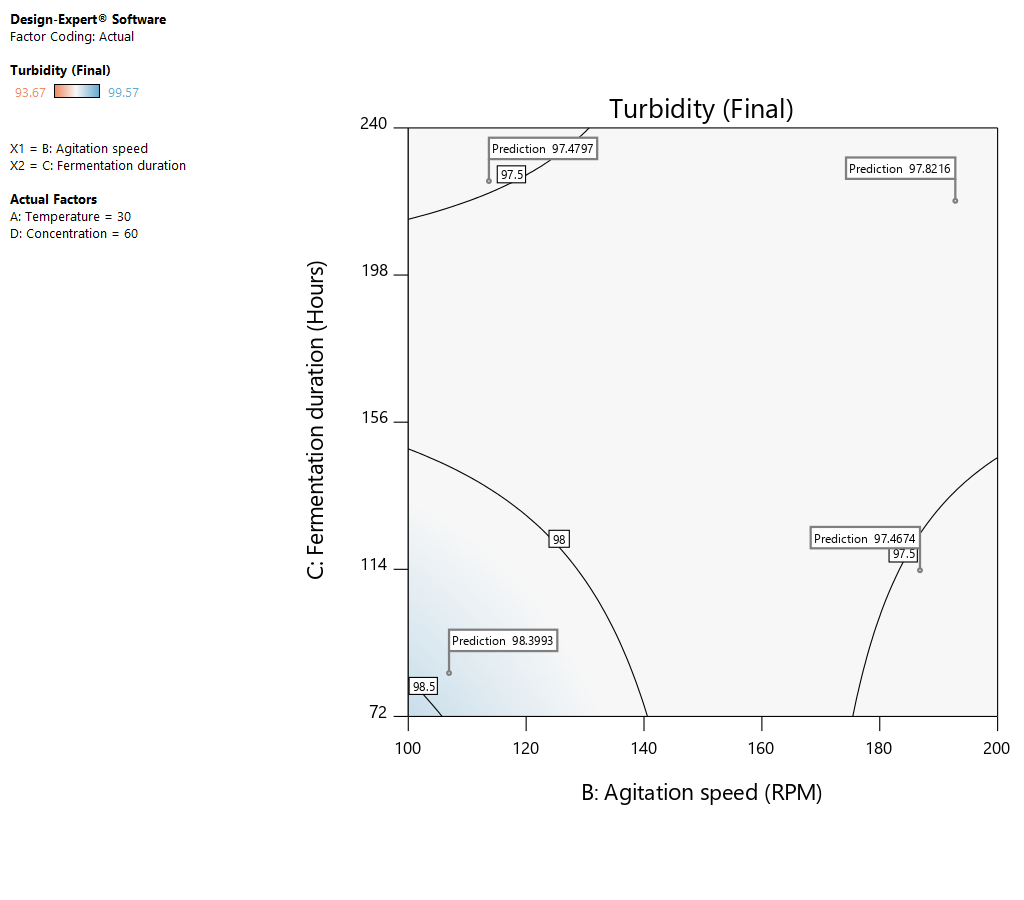 |
| 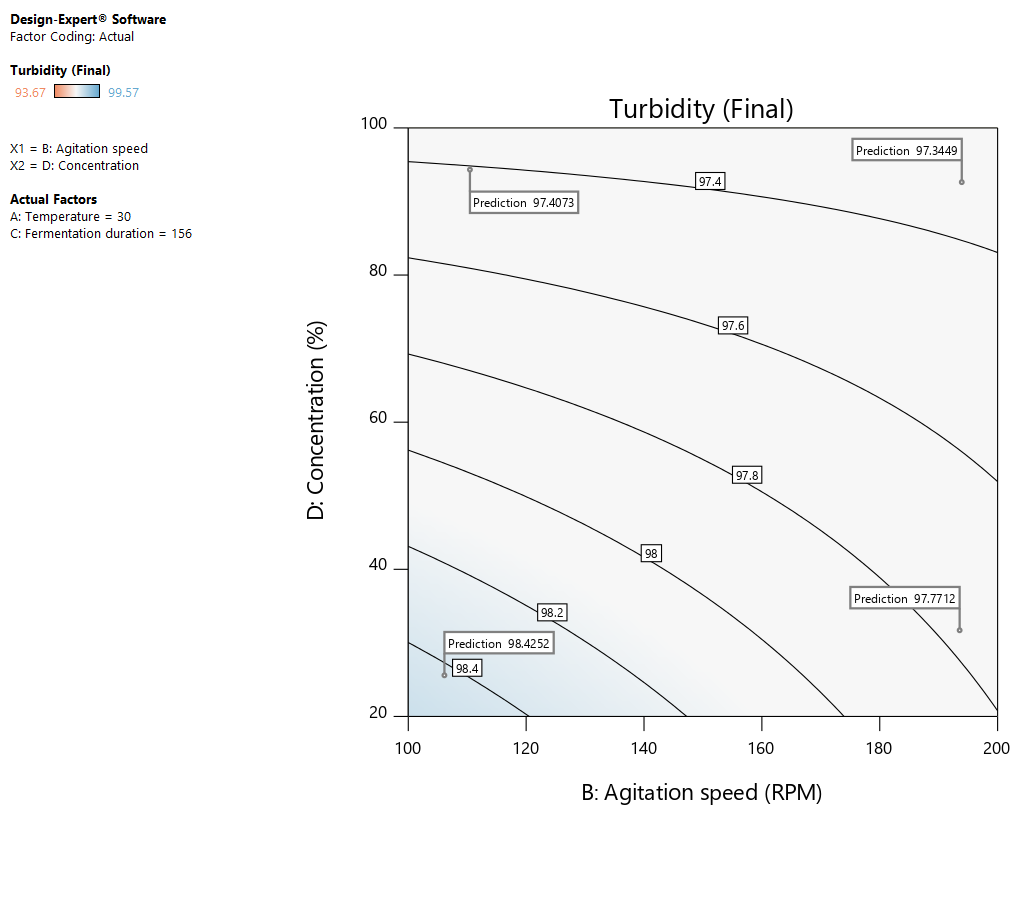 | 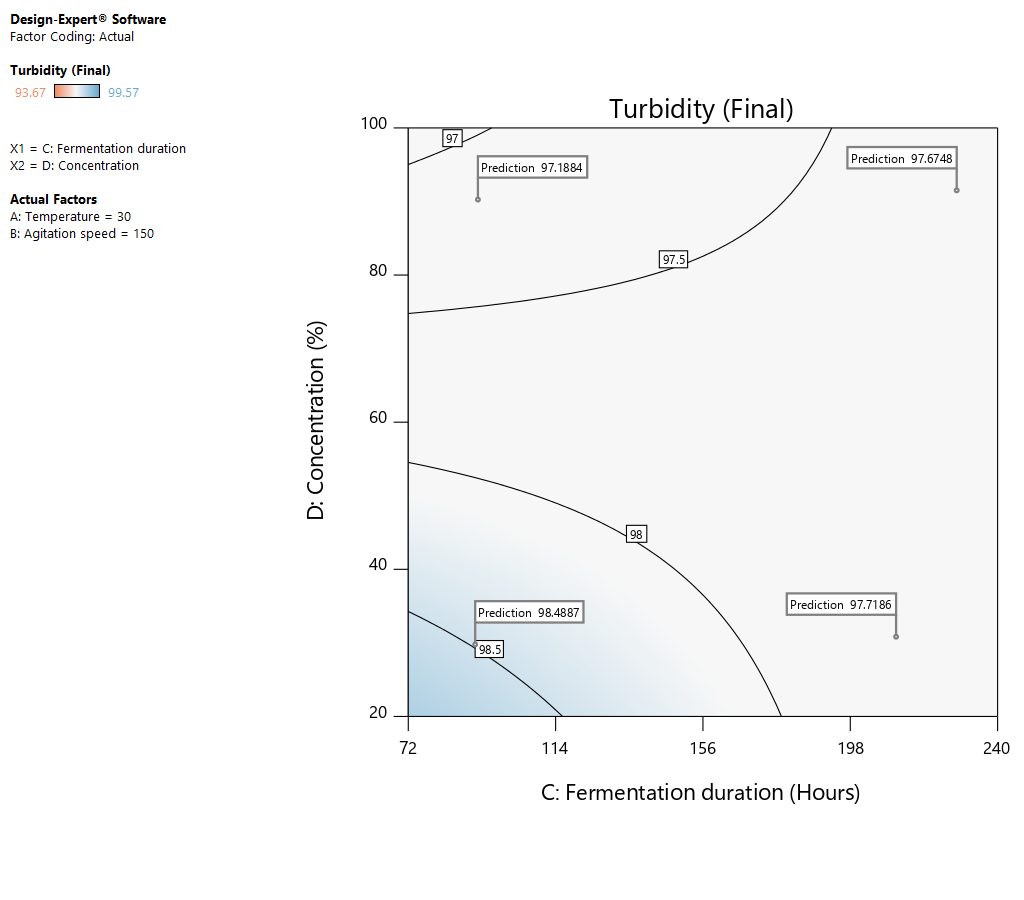 |

Contour plot of the effect of parameters on turbidity removal
